# Associations of urinary fetuin-A with histopathology and kidney events in biopsy-proven kidney disease

**DOI:** 10.1093/ckj/sfae065

**Published:** 2024-03-14

**Authors:** Ming-Tsun Tsai, Wei-Cheng Tseng, Kuo-Hua Lee, Chih-Ching Lin, Shuo-Ming Ou, Szu-yuan Li

**Affiliations:** Division of Nephrology, Department of Medicine, Taipei Veterans General Hospital, Taipei, Taiwan; School of Medicine, College of Medicine, National Yang Ming Chiao Tung University, Taipei, Taiwan; Institute of Clinical Medicine, National Yang Ming Chiao Tung University, Taipei, Taiwan; Division of Nephrology, Department of Medicine, Taipei Veterans General Hospital, Taipei, Taiwan; School of Medicine, College of Medicine, National Yang Ming Chiao Tung University, Taipei, Taiwan; Institute of Clinical Medicine, National Yang Ming Chiao Tung University, Taipei, Taiwan; Division of Nephrology, Department of Medicine, Taipei Veterans General Hospital, Taipei, Taiwan; School of Medicine, College of Medicine, National Yang Ming Chiao Tung University, Taipei, Taiwan; Institute of Clinical Medicine, National Yang Ming Chiao Tung University, Taipei, Taiwan; Division of Nephrology, Department of Medicine, Taipei Veterans General Hospital, Taipei, Taiwan; School of Medicine, College of Medicine, National Yang Ming Chiao Tung University, Taipei, Taiwan; Institute of Clinical Medicine, National Yang Ming Chiao Tung University, Taipei, Taiwan; Division of Nephrology, Department of Medicine, Taipei Veterans General Hospital, Taipei, Taiwan; School of Medicine, College of Medicine, National Yang Ming Chiao Tung University, Taipei, Taiwan; Institute of Clinical Medicine, National Yang Ming Chiao Tung University, Taipei, Taiwan; Division of Nephrology, Department of Medicine, Taipei Veterans General Hospital, Taipei, Taiwan; School of Medicine, College of Medicine, National Yang Ming Chiao Tung University, Taipei, Taiwan; Institute of Clinical Medicine, National Yang Ming Chiao Tung University, Taipei, Taiwan

**Keywords:** biomarker, biopsy-proven kidney diseases, fetuin-A, histopathology, major adverse kidney events

## Abstract

**Background:**

Fetuin-A is implicated in the pathogenesis of vascular calcification in chronic kidney disease (CKD); however, the relationship between fetuin-A, histopathologic lesions and long-term kidney outcomes in patients with various types of kidney disease remains unclear.

**Methods:**

We measured urinary fetuin-A levels in 335 individuals undergoing clinically indicated native kidney biopsy. The expressions of *fetuin-A* mRNA and protein in the kidney were assessed using RNA sequencing and immunohistochemistry. The association of urinary fetuin-A with histopathologic lesions and major adverse kidney events (MAKE), defined as a decline in estimated glomerular filtration rate (eGFR) of at least 40%, kidney failure or death, was analyzed.

**Results:**

Urinary fetuin-A levels showed a positive correlation with albuminuria (r_s_ = 0.67, *P* < .001) and a negative correlation with eGFR (r_s_ = –0.46, *P* < .001). After multivariate adjustment, higher urinary fetuin-A levels were associated with glomerular inflammation, mesangial expansion, interstitial fibrosis and tubular atrophy, and arteriolar sclerosis. Using a 1 transcript per million gene expression cutoff, we found kidney *fetuin-A* mRNA levels below the threshold in both individuals with normal kidney function and those with CKD. Additionally, immunohistochemistry revealed reduced fetuin-A staining in tubular cells of CKD patients compared with normal controls. During a median 21-month follow-up, 115 patients experienced MAKE, and Cox regression analysis confirmed a significant association between elevated urinary fetuin-A and MAKE. This association remained significant after adjusting for potential confounding factors.

**Conclusion:**

Urinary fetuin-A is associated with chronic histological damage and adverse clinical outcomes across a spectrum of biopsy-proven kidney diseases.

KEY LEARNING POINTS **What was known:**

- Although fetuin-A is associated with uremic vascular calcification, its relationship with histopathologic lesions and long-term kidney outcomes in various kidney diseases is unclear.

**This study adds:**

- In individuals with biopsy-confirmed kidney disease, elevated urinary fetuin-A levels are associated with glomerular inflammation, mesangial expansion, interstitial fibrosis, tubular atrophy and arteriolar sclerosis.
- Furthermore, increased urinary fetuin-A is identified as an independent prognostic factor for major adverse kidney events (MAKE).
- The elevated levels of urinary fetuin-A in chronic kidney disease may be attributed to disrupted filtration barriers and impaired tubular uptake.

**Potential impact:**

- Urinary fetuin-A correlates with chronic histopathological lesions and the occurrence of MAKE across various kidney diseases.

## INTRODUCTION

Chronic kidney disease (CKD), affecting over 10% of the global population [1, 2], results in increased morbidity and mortality, diminished quality of life and a substantial financial burden on healthcare systems [3–5]. Therefore, early detection and intervention are crucial for individuals at risk of CKD [6]. Estimated glomerular filtration rate (eGFR) and the spot urine protein-to-creatinine ratio (UPCR) are conventionally used for evaluating kidney function, but they lack sensitivity and specificity for early kidney injury detection [7–9]. As a result, researchers are actively searching for novel kidney biomarkers to accurately assess the presence and severity of kidney disease [10–12]. Some studies suggest that incorporating these biomarkers into the traditional prediction model may enhance prognostic classification of CKD patients, although further validation and characterization are needed [13, 14].

Fetuin-A (alpha2-Heremans-Schmid glycoprotein; AHSG), an abundant multifunctional glycoprotein, is primarily synthesized in the liver [15–17]. Prior research has suggested that fetuin-A may be involved in CKD pathogenesis and its associated cardiovascular complications. By forming soluble complexes with circulating calcium phosphate, fetuin-A can inhibit calcification outside of bone, protecting soft tissues from mineral deposition [18]. Low serum fetuin-A levels are associated with higher cardiovascular and all-cause mortality in kidney failure patients [19], but this is not consistently observed in non-dialysis-dependent CKD patients [20]. Recent evidence even suggests that fetuin-A may promote atherosclerosis by affecting endothelial function, foam cell formation and vascular smooth muscle cell proliferation [21]. Additionally, the role of fetuin-A in the development and progression of CKD is more complex than initially thought. In obese individuals, high circulating fetuin-A levels are associated with downregulated adiponectin–AMPK signaling in podocytes, leading to foot process effacement, albuminuria and decreased eGFR [22]. On the other hand, fetuin-A is a hypoxia-inducible factor (HIF) target gene and supplementation with it immediately after kidney ischemia–reperfusion injury in mice can reduce kidney fibrosis and inflammation severity [23]. The reasons for the discrepancies in these findings are unclear, emphasizing the need for a better understanding of the role of fetuin-A in CKD pathophysiology.

Beyond the existing contradictions, previous studies on the association between fetuin-A and cardiovascular and kidney outcomes have primarily involved patients without kidney biopsies. A recent study found that 29 urinary peptide segments, including fetuin-A, form a classifier that effectively identifies kidney fibrosis in CKD patients [24]. Compared with blood, urine is less invasive and easier to sample repeatedly, and more directly reflects kidney damage and changes [25]. Therefore, this study aimed to evaluate whether urinary fetuin-A could serve as a potential biomarker for kidney histopathology in 335 patients with various biopsy-proven kidney diseases, measuring its levels using enzyme-linked immunosorbent assay (ELISA). To identify the origin of urinary fetuin-A, we analyzed the mRNA and protein levels of kidney fetuin-A in individuals with both normal kidney function and CKD. Furthermore, we investigated the relationship between urinary fetuin-A and the risk of major adverse kidney events (MAKE) to assess its prognostic potential.

## MATERIALS AND METHODS

### Study population

The Taipei Renal Transcriptomics and Outcomes Investigation (TRTOI) is a prospective observational study at Taipei Veterans General Hospital, Taiwan, involving adults (≥20 years old) who underwent kidney biopsies or nephrectomies since October 2018. Its primary goal is to study kidney transcriptomic profiles and discover new circulating biomarkers for CKD. Tissue specimens were processed per standard procedures and interpreted by an experienced nephropathologist [26, 27]. Blood and urine samples were collected on the biopsy day. The study followed the Declaration of Helsinki and was approved by the local ethics committee, with written informed consent from all participants. The exclusion criteria, including individuals who refused consent, kidney transplant recipients, pregnant women and patients with inadequate biopsy samples, led to 335 participants. Supplementary data, Fig. S1 displays the study cohort enrollment flowchart.

### Statement of ethics

This study protocol was reviewed and approved by the Institutional Review Board of Taipei Veterans General Hospital (No. 2018-06-008B). Written informed consent was obtained from each participant.

### Profiling gene expression in the tubulointerstitial compartment of TRTOI patients

A fraction of the kidney biopsy tissue was preserved in RNAlater (Invitrogen) and microdissected to isolate the tubulointerstitial portion. The mRNA expression profile of the tubulointerstitial portion was sequenced using Illumina NovaSeq 6000, generating 150-bp paired-end reads. We performed quality control checks using standard FastQC settings (v0.11.8) and then aligned the processed reads to the human reference genome (GRCh37/hg19) using STAR (Version 2.6.1). RSEM software (Version 1.3.1) was used to calculate transcripts per million (TPM) values, offering a quantitative gene expression assessment. A TPM threshold of 1 indicated gene expression (TPM >1) or its absence (TPM ≤1).

### Urinary fetuin-A measured by ELISA

Fresh urine samples from study participants were collected, centrifuged at 1000*g* for 10 min, and stored at –80°C for analysis. Urinary fetuin-A was measured using a commercial ELISA kit (Catalog no. 30 192 653; Bio Preventive Medicine Corp., Hsinchu, Taiwan) with a detection range of 7.81–500 ng/mL and coefficients of variation less than 10% for intra-assay and 15% for inter-assay. Urinary fetuin-A levels were adjusted for urinary creatinine concentrations, and all measurements were conducted in triplicate for enhanced accuracy.

### Histopathological evaluation

Histopathological lesions were characterized and graded following the method by Srivastava *et al*., including assessments of glomerular, vascular and tubulointerstitial injuries [28]. The distribution of severity grades for the eight histopathologic lesions is detailed in Supplementary data, Table S1. For simplification, endocapillary proliferation, extracapillary cellular crescents, glomerular fibrinoid necrosis and fibrocellular crescents were combined into a dichotomous variable termed “glomerular inflammation” [29]. The final primary clinicopathologic diagnosis was determined by reviewing electronic medical records, laboratory findings and histopathological examinations. Patients were categorized into six diagnostic groups, with the primary clinicopathologic diagnoses presented in Supplementary data, Table S2.

### Clinical information

Baseline patient data included age, sex, body mass index (BMI), comorbidities, current medications (angiotensin-converting enzyme inhibitors, angiotensin II receptor blockers, other antihypertensives, corticosteroids and other immunosuppressants) and laboratory tests. Kidney function was evaluated using eGFR (simplified Modification of Diet in Renal Disease formula), and proteinuria and albuminuria were evaluated through spot UPCR and spot urine albumin-creatinine ratio (UACR).

### Adverse kidney outcomes

MAKE was defined as a composite outcome: a decrease in eGFR of ≥40% from baseline to the last visit, kidney failure, or mortality from kidney or cardiovascular causes, whichever occurred first. The National Kidney Foundation and the Food and Drug Administration recommend a 40% eGFR decline as a broadly acceptable kidney endpoint across various baseline eGFR levels [30]. Participants were followed until death, kidney replacement therapy initiation, loss to follow-up or 31 March 2023. To reduce bias in outcome assessment, we censored observations at 1.5 years from the last eGFR measurement if no subsequent measurement was available within this time frame [31].

### Immunohistochemistry

Formalin-fixed, paraffin-embedded kidney biopsy tissues from 12 CKD patients, including those with diabetic kidney disease (DKD), chronic tubulointerstitial nephritis and nephrosclerosis, as well as 5 healthy controls with thin basement membrane disease, were prepared for immunohistochemistry (IHC) staining. We stained sequential sections with antibodies against fetuin-A (HPA001524, 1:200; Sigma-Aldrich) and megalin (HPA005980, 1:1000; Sigma-Aldrich). Anti-fetuin-A was detected using a goat anti-rabbit horseradish peroxidase (HRP)-conjugated antibody and anti-megalin with an AP-conjugated anti-rabbit antibody. Color reactions used 3,3′-diaminobenzidine (DAB) for fetuin-A and red alkaline phosphatase substrate for megalin, followed by counterstaining the slides with hematoxylin.

### Western blot analysis

Creatinine-normalized urine samples were diluted 10-fold with premixed Laemmli protein sample buffer, separated using SDS-PAGE, and transferred to a polyvinylidene fluoride membrane. These membranes were incubated with an anti-fetuin-A antibody (HPA001524, 1:1000; Sigma-Aldrich), followed by an HRP-conjugated secondary antibody. Protein bands were detected using enhanced chemiluminescence, and band intensity was quantified using ImageJ software (National Institutes of Health, Bethesda, MD, USA).

### Statistical analysis

All variables were complete with no missing data, and data were presented as mean ± standard deviation, median (interquartile range) or number (percentage) as appropriate. Urinary fetuin-A levels were natural log-transformed due to skewed distribution. Individuals were categorized based on tertiles of urinary fetuin-A concentration for relevant parameter assessment. Correlations between clinical-laboratory parameters and fetuin-A levels were assessed using Spearman correlation. Multivariate logistic regression analyses assessed the diagnostic performance of urinary fetuin-A for each histopathological lesion, adjusting for age, sex, albuminuria and eGFR. The cumulative MAKE incidence was assessed using Kaplan–Meier curves and the log-rank test, stratified by urinary fetuin-A tertiles. Subsequently, univariate and multivariate Cox proportional hazards regression analyses were conducted to examine the association between urinary fetuin-A and adverse kidney outcomes. The multivariate model was adjusted for baseline covariates, including age, sex, hemoglobin, eGFR, albuminuria, renin–angiotensin–aldosterone system inhibitors, glucocorticoid and non-glucocorticoid immunosuppressive agents, as well as the primary clinicopathologic diagnosis (DKD vs other diagnoses). The performance of urinary fetuin-A and UACR in prognosticating clinical outcomes was evaluated by constructing receiver operating characteristic (ROC) curves. The optimal cutoff point and the maximum summation value of sensitivity and specificity were determined using the Youden index. The areas under the curve (AUC) of each examination were compared using DeLong's test. All statistical tests were two-tailed with a significance level of *P* < .05, using SAS version 9.4 (SAS Institute, Inc., Cary, NC, USA) and R software 3.5.2 (R Development Core Team, Vienna, Austria, 2018).

## RESULTS

### The baseline characteristics of the study cohort

Baseline patient characteristics are summarized in Table 1. The cohort had a mean age of 53 ± 16 years, with 57% men and 28% having diabetes. Median eGFR and albuminuria were 40 (19–78) mL/min/1.73 m^2^ and 2.4 (0.7–5.0) mg/mg creatinine, respectively. Proliferative glomerulonephritis, nonproliferative glomerulopathies and DKD were the most common kidney disease types, accounting for 32%, 26% and 19% of cases, respectively. The median baseline urinary fetuin-A concentration was 57 (21–170) ng/mg creatinine. Supplementary data, Table S3 shows kidney function tests and urinary fetuin-A levels categorized by primary clinicopathologic diagnostic groups. DKD patients had higher UACR and urinary fetuin-A levels, along with lower eGFRs, compared with other kidney disease groups.

**Table 1:** The baseline characteristics of study participants in the TRTOI cohort (*N* = 335).

Parameters	
Urinary fetuin-A concentrations, ng/mg creatinine	57 (21–170)
Demographic and clinical characteristics	
Age, years	53 ± 16
Male, *n* (%)	192 (57)
BMI, kg/m^2^	25.5 ± 4.5
Diabetes mellitus, *n* (%)	95 (28)
Hypertension, *n* (%)	139 (42)
Prevalent CVDs, *n* (%)	40 (12)
Laboratory test results	
Hemoglobin, g/dL	11.3 (9.4–13.3)
Leukocytes, 10^3^/mm^3^	6.7 (5.3–8.1)
Albumin, g/dL	3.2 (2.6–3.9)
BUN, mg/dL	27 (17–46)
Creatinine, mg/dL	1.6 (0.9–3.2)
eGFR, mL/min/1.73 m^2^	40 (19–78)
Proteinuria, mg/mg creatinine	3.1 (1.1–7.3)
Albuminuria, mg/mg creatinine	2.4 (0.7–5.0)
Clinicopathologic diagnostic categories, *n* (%)	
Proliferative glomerulonephritis	106 (32)
Nonproliferative glomerulopathies	86 (26)
DKD	62 (19)
Vascular	26 (8)
Tubulointerstitial	23 (7)
Other	32 (10)
Medications, *n* (%)	
ACEi/ARB	197 (59)
Beta-blockers	148 (44)
Calcium channel blockers	198 (59)
Glucocorticoids	186 (56)
Immunosuppressants other than glucocorticoids	64 (19)

Data are presented as mean ± standard deviation, median (interquartile range) or count with percentages.

ACEi, angiotensin-converting enzyme inhibitors; ARB, angiotensin II type 1 receptor blockers; BUN, blood urea nitrogen; CVDs, cardiovascular diseases.

We conducted a further analysis of correlations between urinary fetuin-A levels, clinicodemographics and laboratory measurements (Table 2). Urinary fetuin-A levels were negatively correlated with hemoglobin, serum albumin and eGFR (r_s_ = –0.32, –0.40, –0.46; all *P* < .001) and positively correlated with age, BMI, leukocyte counts and UACR (r_s_ = 0.16, 0.13, 0.12, 0.67; *P* = .004, .02, .03, <.001).

**Table 2:** Spearman correlation coefficients between clinical characteristics, laboratory data and urinary fetuin-A.

	uFetA	Age	BMI	WBC	Hgb	sAlb	eGFR	UACR
uFetA	1	0.16 (.004)	0.13 (.02)	0.12 (.03)	–0.32 (<.001)	–0.40 (<.001)	–0.46 (<.001)	0.67 (<.001)
Age	0.16 (.004)	1	–0.10 (.07)	–0.06 (.25)	–0.17 (.002)	–0.06 (.31)	–0.30 (<.001)	0.06 (.30)
BMI	0.13 (.02)	–0.10 (.07)	1	0.07 (.19)	0.14 (.01)	–0.18 (.001)	0.05 (.39)	0.23 (<.001)
WBC	0.12 (.03)	–0.06 (.25)	0.07 (.19)	1	0.03 (.64)	–0.04 (.45)	–0.15 (.006)	0.07 (.21)
Hgb	–0.32 (<.001)	–0.17 (.002)	0.14 (.01)	0.03 (.64)	1	–0.01 (.89)	0.57 (<.001)	–0.10 (.07)
sAlb	–0.40 (<.001)	–0.06 (.31)	–0.18 (.001)	–0.04 (.45)	–0.01 (.89)	1	–0.16 (.003)	–0.71 (<.001)
eGFR	–0.46 (<.001)	–0.30 (<.001)	0.05 (.39)	–0.15 (.006)	0.57 (<.001)	–0.16 (.003)	1	–0.08 (.13)
UACR	0.67 (<.001)	0.06 (.30)	0.23 (<.001)	0.07 (.21)	–0.10 (.07)	–0.71 (<.001)	–0.08 (.13)	1

*P*-values are in parentheses.

uFetA, urinary fetuin-A; Hgb, hemoglobin; sAlb, serum albumin; uFetA, urinary fetuin-A concentrations (ng/mg creatinine); WBC, white blood cell count.

### The associations of urinary fetuin-A with kidney histopathologic lesions

We examined the relationship between urinary fetuin-A and kidney histological lesions. In Table 3, we presented multivariable-adjusted odds ratios and 95% confidence intervals for these histopathological features based on both continuous and categorical urinary fetuin-A levels. We observed that higher urinary fetuin-A levels were associated with glomerular inflammation, mesangial expansion, interstitial fibrosis/tubular atrophy (IFTA) and arteriolar sclerosis. Furthermore, patients in the highest urinary fetuin-A tertile were more likely to have glomerular inflammation, mesangial expansion, IFTA, arteriosclerosis and arteriolar sclerosis compared with those in the lowest tertile.

**Table 3:** The association between urinary fetuin-A and each histopathological lesion.

	OR^a^ (95% CI)							
Variables	GI^b^	ME^b^	SS^b^	GS^c^	ATI^b^	IFTA^d^	Arteriosclerosis^e^	Arteriolosclerosis^e^
uFetA (ng/mg Cre)								
Tertile 1	Reference	Reference	Reference	Reference	Reference	Reference	Reference	Reference
Tertile 2	1.46 (0.77–2.76)	1.34 (0.76–2.35)	2.31 (1.29–4.12)	1.15 (0.57–2.30)	1.30 (0.70–2.41)	1.33 (0.58–3.02)	1.44 (0.80–2.61)	1.31 (0.60–2.89)
Tertile 3	2.47 (1.11–5.50)	3.32 (1.63–6.80)	1.81 (0.91–3.59)	1.41 (0.65–3.05)	0.63 (0.29–1.36)	2.62 (1.01–6.77)	2.19 (1.09–4.41)	3.27 (1.46–7.33)
Per 1-Ln increase	1.26 (1.01–1.58)	1.27 (1.05–1.53)	1.19 (0.98–1.44)	1.06 (0.86–1.32)	0.93 (0.76–1.15)	1.31 (1.01–1.71)	1.17 (0.96–1.43)	1.44 (1.13–1.83)

a Logistic regression models were constructed using each histopathological lesion as the dependent variable and the tertiles and Ln-transformed urinary fetuin-A as the independent variables. Each multivariate model was adjusted for age, sex, albuminuria and eGFR.

b Dependent variable is presence of lesion.

c Dependent variable is involvement of >25% of glomeruli.

d Dependent variable is involvement of >25% of cortical volume.

e Dependent variable is moderate-to-severe degree of lesion severity.

ATI, acute tubular injury; CI, confidence interval; Cre, creatinine; GI, glomerular inflammation; GS, global glomerulosclerosis; Ln, natural logarithm; ME, mesangial expansion; OR, odds ratio; SS, segmental glomerulosclerosis; uFetA, urinary fetuin-A.

### The potential origin of urinary fetuin-A in CKD patients

**Figure 1:**
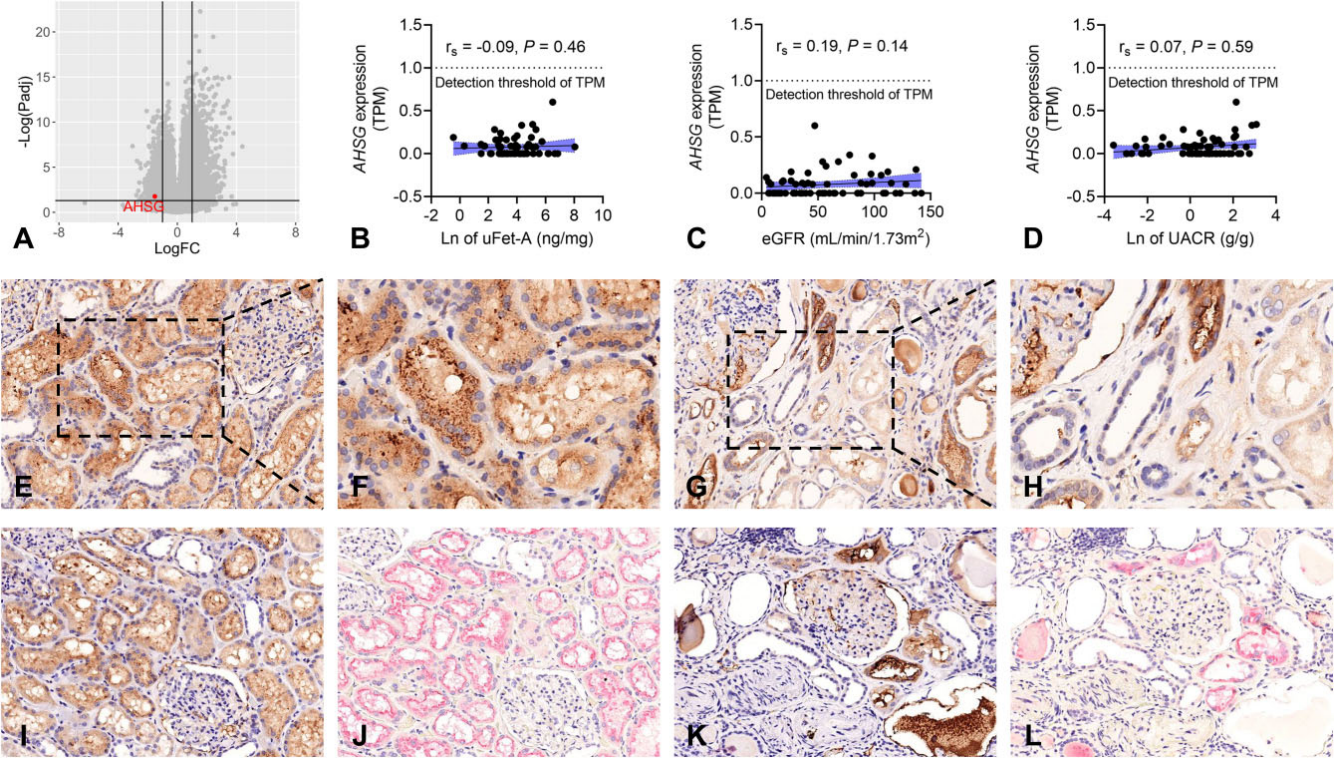
Studying fetuin-A expression in kidneys of healthy individuals and CKD patients. (**A**) The volcano plot highlights *AHSG* downregulation in fibrotic kidney disease compared with non-fibrotic cases. X-axis shows Log2 fold change, Y-axis shows -Log10 (*P*-values). (B–D) Correlation between *AHSG* transcript levels (TPM), urinary fetuin-A levels (**B**), eGFR (**C**) and albuminuria (**D**) in 64 microdissected human tubulointerstitial samples using a 1 TPM threshold to define gene expression. (E–H) IHC staining of fetuin-A in kidney biopsies from CKD patients and normal controls. Fetuin-A is prominently expressed in normal kidney proximal tubular cells (**E**), reduced in advanced CKD (**G**). Enlarged views of (**E**) and (**G**) in (**F**) and (**H**). (I–L) IHC staining for fetuin-A and megalin in sequential paraffin-embedded kidney sections. Fetuin-A and megalin colocalization in tubules is evident in normal controls (**I, J**), whereas in CKD, both fetuin-A (**K**) and megalin-positive staining (**L**) are absent in dilated and atrophic tubules. (Original magnifications ×200 (E, G and I–L) and ×400 (F and H)]. Ln, natural logarithm; uFetA, urinary fetuin-A concentrations (ng/mg creatinine).

We conducted additional research to determine whether the injured kidney is the primary source of elevated urinary fetuin-A in CKD. RNA sequencing (RNA-seq) was conducted on tubulointerstitial compartments from the initial 64 participants, and their clinical characteristics, along with those without RNA-seq, are presented in Supplementary data, Table S4. Using an IFTA threshold of >10% to define kidney fibrosis, *AHSG*, the gene encoding fetuin-A, significantly decreased in individuals with fibrotic kidneys (*n* = 40) compared with non-fibrotic samples (*n* = 24) (Fig. 1A). Kidney *AHSG* mRNA levels were below the TPM threshold of 1 in both CKD patients and those with normal kidney function, with no correlation to urinary fetuin-A, eGFR or UACR (Fig. 1B–D). Additionally, a publicly available single-nucleus RNA-seq dataset from adult human kidneys showed *AHSG* expression in 2%–2.5% of the loop of Henle and distal convoluted tubules in healthy controls [32], but a decrease in DKD patients (Supplementary data, Fig. S2). These findings collectively suggest that the kidney does not directly produce urinary fetuin-A in CKD.

We also performed IHC to investigate the localization and expression of fetuin-A within the kidney. Normal proximal tubules displayed robust and widespread fetuin-A staining. In contrast, CKD patients exhibited limited fetuin-A staining in tubular cells, possibly due to reduced proximal tubular endocytic capabilities (Fig. 1E–H). Further support for this was provided by immunostaining of sequential kidney biopsy sections, which demonstrated the co-localization of fetuin-A with the endocytic receptor megalin in healthy individuals’ tubules (Fig. 1I and J). However, in CKD patients, megalin expression was reduced in proximal tubules, with no detectable fetuin-A staining in these tubular epithelia (Fig. 1K and L).

The potential rise in urine fetuin-A levels due to proteolytic degradation is suggested [33], supported by the presence of lower molecular weight bands in the urine of CKD patients compared with healthy controls (Supplementary data, Fig. S3). In urine samples from CKD patients, there was not only increased urinary fetuin-A at 58 kDa but also a band at approximately 39 kDa, possibly indicating a proteolytic fragment of fetuin-A. Supplementary data, Table S5 includes clinical details for individuals whose urine underwent western blot analysis.

### Associations of urinary fetuin-A with MAKE

**Figure 2:**
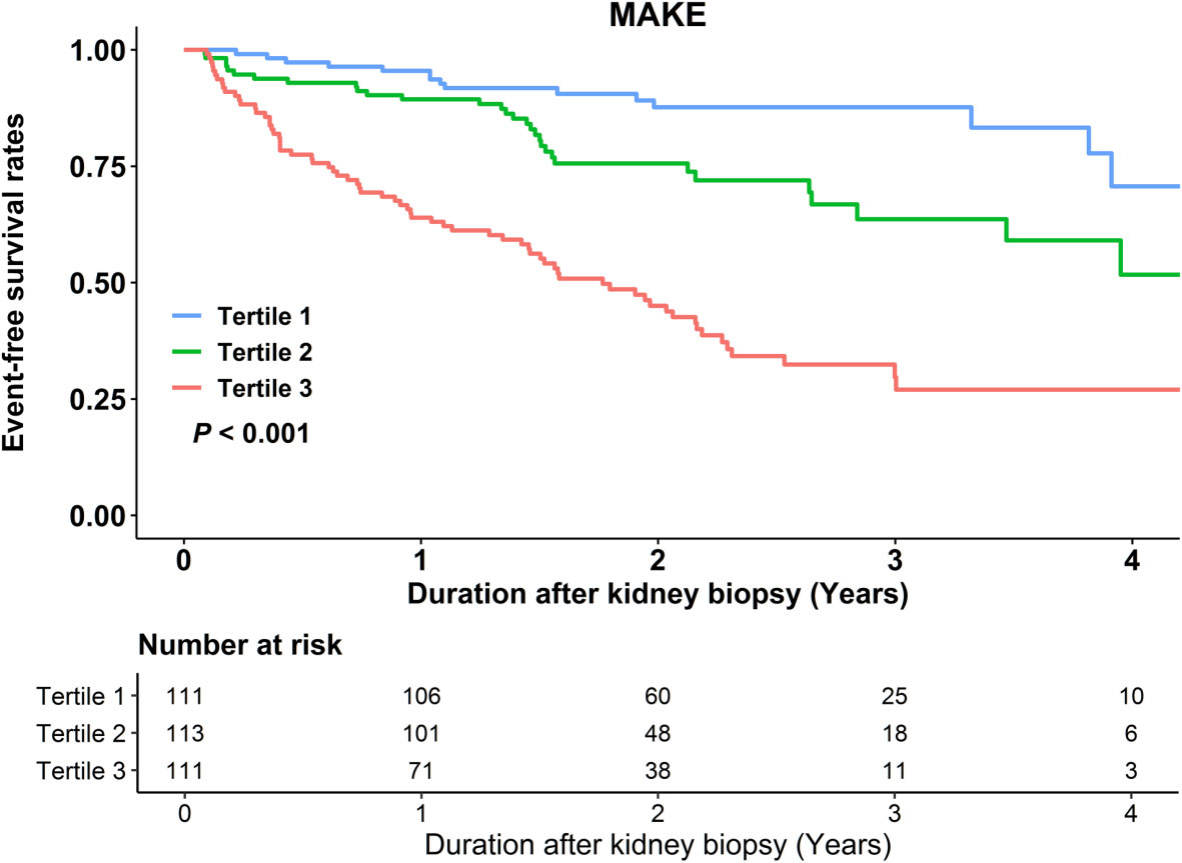
Cumulative kidney outcome incidence based on urinary fetuin-A tertiles. In kidney biopsy patients, the 2-year MAKE rates were 10% for tertile 1 [urinary fetuin-A: 12 (6–21) ng/mg creatinine], 21% for tertile 2 [urinary fetuin-A: 57 (40–87) ng/mg creatinine] and 52% for tertile 3 [urinary fetuin-A: 266 (170–495) ng/mg creatinine]. Kaplan–Meier survival analysis, compared using the log-rank test, revealed significant differences in kidney outcomes.

Over a median 21 (14–32) months of follow-up, 28 participants were lost to follow-up, and 115 patients experienced MAKE, which included death (*n* = 20), kidney failure (*n* = 64) and kidney function decline (*n* = 31). Figure 2 displays Kaplan–Meier curves for cumulative MAKE incidence based on urinary fetuin-A tertiles. The high fetuin-A group is associated with a higher risk of MAKE compared with the low fetuin-A group (*P* < .001, log-rank test). Table 4 shows univariable and multivariable-adjusted HRs for urinary fetuin-A, both categorical and continuous variables, in relation to MAKE. We found a significant association between elevated urinary fetuin-A and adverse kidney outcomes, even after adjusting for potential confounding factors. Furthermore, the prognostic performance of urinary fetuin-A concentration and UACR for the development of MAKE was assessed and compared using ROC curves (shown in Supplementary data, Fig. S4 and Table S6). We found that the AUC of urinary fetuin-A for predicting MAKE was 0.75 (95% confidence interval 0.70–0.80, sensitivity = 64%, specificity = 79%), which was significantly superior to that of UACR.

**Table 4:** Associations of urinary fetuin-A with MAKE.

	Model 1	Model 2	Model 3
Variables	HR (95% CI)	HR (95% CI)	HR (95% CI)
uFetA (ng/mg Cre)			
Tertile 1: 12 (6–21)^a^	Reference	Reference	Reference
Tertile 2: 57 (40–87)^a^	2.32 (1.25–4.30)	1.61 (0.85–3.03)	1.62 (0.86–3.04)
Tertile 3: 266 (170–495)^a^	6.64 (3.79–11.63)	2.45 (1.30–4.60)	1.92 (1.04–3.57)
Per 1-Ln increase	1.68 (1.47–1.92)	1.39 (1.17–1.65)	1.23 (1.03–1.47)

a Median (interquartile range).

Model 1 is unadjusted.

Model 2 includes adjustments for age, sex, hemoglobin, baseline albuminuria, renin–angiotensin–aldosterone system inhibitors (angiotensin-converting enzyme inhibitors or angiotensin receptor blockers), immunosuppressive medications (glucocorticoids or other immunosuppressants) and primary clinicopathologic diagnosis.

Model 3 is Model 2 with additional adjustment for baseline eGFR.

HR, hazard ratio; CI, confidence interval; uFetA, urinary fetuin-A; Ln, natural logarithm.

## DISCUSSION

Fetuin-A plays a crucial role in various biological processes, including those in the kidney. The use of urinary fetuin-A as a prognostic marker for CKD patients has been limited in previous research. Although some groups have suggested a link between high urinary fetuin-A levels and a faster eGFR decline rate [34–36], none explored its relationship with specific kidney pathology. In our study of 335 individuals who underwent kidney biopsies, we found that urinary fetuin-A is independently associated with an adverse kidney prognosis. Moreover, a robust correlation between fetuin-A levels and chronic kidney damage on biopsy emerged, highlighting its potential as a noninvasive biomarker for CKD histopathological assessments.

Elevated urinary fetuin-A levels in individuals with impaired kidney function can be attributed to several mechanisms. This negatively charged plasma protein, with a molecular weight ranging from 51 to 67 kDa, faces challenges passing through normal glomerular capillaries [37]. In cases of glomerular disorders, disrupted filtration barriers result in increased protein leakage into the urine [38]. Furthermore, upregulated proteases specific to cleaving fetuin-A in the kidneys of patients with proteinuric kidney disease enable its passage through the glomerulus [35, 39, 40]. Our western blot analysis detected a 39 kDa band in CKD urines, indicating the presence of low molecular weight fetuin-A forms. Additionally, like other proteins such as retinol binding protein, alpha-1 microglobulin or light chains, fetuin-A is also reabsorbed and degraded by megalin [41, 42]. Therefore, the reduced expression of megalin in proximal kidney tubules may potentially increase urinary fetuin-A levels in CKD [43]. Our study showed a significant correlation between urinary fetuin-A levels and albuminuria, mainly due to disrupted filtration barriers in most cases of glomerular disease. Additionally, the reduced co-localized staining of fetuin-A and megalin in affected kidney tubules may emphasize the significance of impaired tubular uptake in the elevated levels of urinary fetuin-A in CKD.

Urinary fetuin-A levels may also depend on liver production. Numerous studies have demonstrated a significant increase in serum fetuin-A levels in patients with nonalcoholic fatty liver disease (NAFLD), which is associated with insulin resistance and an elevated risk of future diabetes compared with controls [44, 45]. An increase in serum levels of fetuin-A may lead to higher excretion of fetuin-A into urine. Additionally, glucagon-like peptide-1 (GLP-1) receptor agonists, known for their greater impact on weight loss compared with other antihyperglycemic agents, have been reported to reduce hepatic fat content and circulating fetuin-A concentrations in patients with type 2 diabetes mellitus and NAFLD [46]. These factors may explain the observed phenomenon in this study, where patients with DKD exhibit higher urinary fetuin-A concentrations compared with other kidney disease patients, beyond factors related to kidney damage itself.

While fetuin-A is recognized as a hepatokine, it can also be produced in adipocytes, monocytes/macrophages and other cells [47]. The potential production of fetuin-A in the kidney following injury remains uncertain. Piazzon *et al*. found increased kidney fetuin-A expression in mouse models of autosomal dominant polycystic kidney disease (ADPKD) [34], and another study demonstrated that hypoxia can induce fetuin-A expression in cultured kidney tubular cells and the kidneys of mice with fetal growth restriction [23]. In contrast, our bulk RNA-seq analysis found minimal *AHSG* mRNA in kidney biopsy tissues, indicating that the kidney may not be a significant source of urinary fetuin-A in both healthy individuals and CKD patients.

The severity of chronic histopathologic lesions independently predicts CKD progression, even when accounting for traditional risk factors [28]. Our study found elevated urinary fetuin-A levels associated with chronic kidney pathology, potentially linking fetuin-A to poor kidney outcomes. However, whether urinary fetuin-A is merely an innocent bystander or an active participant in CKD progression and kidney inflammation remains debated. Elevated urinary fetuin-A levels have been reported as a surrogate marker for insulin resistance and systemic inflammation in obesity and type 2 diabetes, both key contributors to CKD development [22]. Additionally, blocking receptor-mediated endocytosis with a megalin inhibitor retained fetuin-A in the proximal tubule, protecting the kidneys from nephrocalcinosis in parathyroid hormone-treated rats [41]. Another study showed that knocking out fetuin-A expression in a mouse model of hypoxia-induced intrauterine growth restriction exacerbated kidney function decline and tubulointerstitial fibrosis [23]. These findings suggest that fetuin-A may directly impact kidney pathophysiology, although the effects of excess urinary fetuin-A on injured tubular cells in CKD patients require further investigation.

Urinary fetuin-A, beyond its prognostic value in advanced CKD, addresses the limitations of eGFR and proteinuria, enabling early CKD detection. Recent research shows that it rises earlier than albuminuria in diabetic patients with subsequent kidney function decline, supporting early subclinical kidney disease detection [35]. Additionally, fetuin-A can help determine the underlying cause of kidney disease, with one study achieving moderate success in differentiating ADPKD from other kidney diseases and healthy controls [34]. Overall, available data suggests urinary fetuin-A levels can be valuable for diagnosing and prognosticating kidney disease severity, in both early and advanced CKD.

Several study limitations deserve mention. First, plasma fetuin-A was not quantified, preventing a comparison of its prognostic value with urinary fetuin-A in these patients. Second, despite adjusting for potential confounders, unmeasured factors may influence the estimated impact of urinary fetuin-A on the risk of adverse kidney outcomes. Third, the small sample sizes within diagnostic categories precluded additional subgroup analysis. Lastly, urinary fetuin-A levels may vary depending on the type of ELISA used [48]. As such, further validation of these findings in an independent CKD patient cohort is warranted.

In conclusion, our study found that fetuin-A is negatively associated with eGFR and positively associated with albuminuria. Additionally, urinary fetuin-A is linked to chronic histopathological lesions and adverse clinical outcomes in patients undergoing kidney biopsy, suggesting its potential value for clinical decision-making in this population.

## Supplementary Material

sfae065_Supplemental_File

## Data Availability

All data generated or analyzed during this study are included in this article. Further enquiries can be directed to the corresponding author.
