# Supplementary material for: Associations of urinary fetuin-A with histopathology and kidney events in biopsy-proven kidney disease
Source: Clin Kidney J. 2024 Mar 14;17(4):sfae065. doi: 10.1093/ckj/sfae065 (PMC10993056; doi:10.1093/ckj/sfae065)
Supplement: sfae065_Supplemental_File [file sfae065_supplemental_file.pdf]

Supplementary Information

Associations of Urinary Fetuin-A with Histopathology and Kidney Events in Biopsy-Proven Kidney Disease

Ming-Tsun Tsai, MD, PhD,[1,2,3] Wei-Cheng Tseng, MD, PhD,[1,2,3] Kuo-Hua Lee, MD,[1,2,3] Chih-Ching Lin, MD, PhD,[1,2,3] Shuo-Ming Ou, MD, PhD,[1,2,3,*] and Szu-yuan Li, MD, PhD[1,2,3,*]

[1]Division of Nephrology, Department of Medicine, Taipei Veterans General Hospital, Taipei, Taiwan.

[2]School of Medicine, College of Medicine, National Yang Ming Chiao Tung University, Taipei, Taiwan.

[3]Institute of Clinical Medicine, National Yang Ming Chiao Tung University, Taipei, Taiwan.

Corresponding Author[*]:

Szu-yuan Li, MD, PhD

Division of Nephrology, Department of Medicine

Taipei Veterans General Hospital

201, Section 2, Shih-Pai Road, Taipei 11217, Taiwan

Email: syli@vghtpe.gov.tw

Or

Shuo-Ming Ou, MD, PhD

Division of Nephrology, Department of Medicine

Taipei Veterans General Hospital

201, Section 2, Shih-Pai Road, Taipei 11217, Taiwan

Email: okokyytt@gmail.com

Supplemental Data, containing 6 tables and 4 figures.

Supplementary Tables

Table S1. Distribution of semiquantitative grading for individual histopathological lesions in The Taipei Renal Transcriptomics and Outcomes Investigation.

Histopathological feature	Grades	Number (percentage)
Glomerular inflammation	0 (absence)	247 (74)
	1 (presence)	88 (26)
Mesangial expansion	0 (absence)	129 (39)
	1 (presence)	206 (62)
Segmental sclerosis	0 (absence)	196 (59)
	1 (presence)	139 (42)
Global glomerulosclerosis	0 (≤10%)	113 (34)
	1 (11-25%)	82 (25)
	2 (26-50%)	90 (27)
	3 (>50%)	50 (15)
Acute tubular injury	0 (absence)	245 (73)
	1 (presence)	90 (27)
Interstitial fibrosis/tubular atrophy	0 (≤10%)	113 (34)
	1 (11-25%)	64 (19)
	2 (26-50%)	103 (31)
	3 (>50%)	55 (16)
Arterial sclerosis	0 (none or mild)	168 (50)
	1 (moderate or severe)	167 (50)
Arteriolar sclerosis	0 (none or mild)	248 (74)
	1 (moderate or severe)	87 (26)

The severity of global glomerulosclerosis and interstitial fibrosis/tubular atrophy is graded by assessing the percentage of affected glomeruli or kidney cortical volume.

Table S2. Primary clinicopathologic diagnoses

PGN, n = 106	NPGP, n = 86	DKD, n = 62	Vascular, n = 26	TI, n = 23	Other, n = 32
IgAN (41)<br><br>MesPGN (8)<br><br>C3 GN (2)<br><br>FGN (2)<br><br>DDD (1)<br><br>LN<br>• Class 2 (2)<br>• Class 3 (A or A/C) (8)<br>• Class 3+5 (A or A/C) (6)<br>• Class 4 (A or A/C) (10)<br>• Class 4+5 (A or A/C) (7)<br><br>MPGN (1)<br><br>AAV (10)<br><br>ICGN (5)<br><br>IgA-IRGN (3)	MCD (30)<br><br>MN (40)<br><br>Idiopathic FSGS (15)<br><br>LN<br>• Class 5 (1)	DKD (62)	NSC (15)<br><br>TMA (10)<br><br>MHT (1)	AIN (6)<br><br>ATN (5)<br><br>Oxalate nephropathy (3)<br><br>CIN (9)	Secondary FSGS (6)<br><br>Minor abnormalities (4)<br><br>Cast nephropathy (2)<br><br>HCDD (1)<br><br>AL amyloid (5)<br><br>Fabry disease (3)<br><br>LCPT (1)<br><br>TBM (5)<br><br>Ischemic changes in glomeruli (2)<br><br>Mild mesangial proliferation (2)<br><br>Idiopathic nodular sclerosis (1)

The number of patients is shown in parentheses.

Abbreviations: A, active lesions; AAV, ANCA associated vasculitis; AIN, acute interstitial nephritis; AL, amyloid light chain; ATN, acute tubular

necrosis; C, chronic lesions; C3 GN; C3 glomerulonephritis; CIN, chronic interstitial nephritis; DDD, dense deposit disease; DKD, diabetic kidney disease; FGN, fibrillary glomerulonephritis; FSGS, focal segmental glomerulosclerosis; HCDD, heavy-chain deposition disease; ICGN, immune-complex glomerulonephritis; IgA-IRGN, IgA-dominant infection-related glomerulonephritis; IgAN, IgA nephropathy; LCPT, light chain proximal tubulopathy; LN, lupus nephritis; MCD, minimal change disease; MesPGN, mesangioproliferative glomerulonephritis; MHT, malignant hypertension; MN, membranous nephropathy; MPGN, membranoproliferative glomerulonephritis; NPGP, non-proliferative glomerulopathies; NSC, nephrosclerosis; PGN, proliferative glomerulonephritis; TBM, thin basement membrane; TI, tubulointerstitial; TMA, thrombotic microangiopathy.

Table S3. Kidney function, proteinuria, and urinary fetuin-A levels by primary clinicopathologic diagnosis

	PGN	NPGP	DKD	Vascular	TI	Other	*P* Value
eGFR, ml/min/1.73m^2	51 (21—87)	79 (47—102)	19 (13—30)	24 (16—37)	20 (13—36)	42 (27—97)	<0.001
Albuminuria, mg/mg Cre	1.5 (0.5—3.3)	3.8 (1.7—8.0)	4.8 (2.7—6.6)	2.2 (1.1—3.6)	0.2 (0.0—0.7)	0.4 (0.0—2.6)	<0.001
uFetA, ng/mg Cre	34.3 (16.2—117.1)	55.5 (15.8—145.8)	210.2 (112.6—366.0)	109.1 (29.6—236.2)	35.8 (19.3—66.1)	34.3 (6.2—82.3)	<0.001

Data presented as median [interquartile range]

Abbreviations: Cre, creatinine; DKD, diabetic kidney disease; eGFR, estimated glomerular filtration rate; NPGN, nonproliferative glomerulopathies; PGN, proliferative glomerulonephritis; TI, tubulointerstitial; uFetA, urinary fetuin-A.

Table S4. Comparison of the characteristics between individuals with and without tubulointerstitial compartment RNA-seq.

	Individuals whose tissue underwent RNA-seq (n=64)	Individuals whose tissue did not undergo RNA-seq (n=271)	*P* value
Age (yr)	56±16	53±17	0.15
Male sex [n (%)]	37 (58)	155 (57)	0.93
eGFR (ml/min/1.73 m^2)	49 (20−92)	38 (19−73)	0.25
UPCR (mg/mg)	2.2 (1.2−6.1)	3.2 (1.0−7.4)	0.41
uFetA (ng/mg Cre)	45 (16−143)	62 (23−181)	
Severity of IFTA [n (%)]			0.23
Minimal (≤10%)	24 (38)	89 (33)	
Mild (11-25%)	16 (25)	48 (18)	
Moderate (26-50%)	18 (28)	85 (31)	
Severe (>50%)	6 (9)	49 (18)	

The data were presented as mean ± standard deviation, median, or number (percentage) as needed.

Abbreviations: eGFR, estimated glomerular filtration rate; IFTA, interstitial fibrosis and tubular atrophy; uFetA, urinary concentration of fetuin-A; UPCR, urine protein to creatinine ratio.

Table S5. Characteristics of study participants in the western blot analysis

Participants	Age/gender	Diagnosis	SCr (mg/dl)	BUN (mg/dl)	eGFR(ml/min/1.73m^2)	UPCR (mg/mg)	uFetA (ng/mg Cre)
1	40/M	HC	0.84	12	101	0.05	3.2
2	38/M	HC	0.89	12	96	0.06	3.2
3	39/M	HC	0.89	18	95	0.07	8.4
4	37/F	TBM	0.76	14	86	0.42	3.3
5	22/M	MesPGN	0.81	11	119	1.35	4.4
6	47/F	MN	0.65	8	98	1.70	5.5
7	51/M	LN	1.97	46	36	4.32	147.5
8	48/M	DKD	1.48	20	51	7.11	274.8
9	54/M	ICGN	2.01	23	35	4.98	136.7

Abbreviations: BUN, blood urea nitrogen; Cre, creatinine; DKD, diabetic kidney disease; eGFR, estimated glomerular filtration rate; F, female; HC, healthy controls; ICGN, immune-complex glomerulonephritis; LN, lupus nephritis; M, male; MesPGN, mesangioproliferative glomerulonephritis; MN, membranous nephropathy; SCr, serum creatinine; TBM, thin basement membrane; uFetA, urinary fetuin-A; UPCR, urine protein-to-creatinine ratio.

Table S6. The prognostic performance of the urinary fetuin-A/creatinine ratio and albuminuria in identifying the occurrence of major adverse kidney events in individuals with biopsy-proven kidney disease.

	**AUC (95% CI)**	***P*-value**	**SEN[a] (%)**	**SPEC[a] (%)**	**PPV[a] (%)**	**NPV[a] (%)**	**PLR[a]**	**NLR[a]**
uFetA (ng/mg Cre)	0.75 (0.70－0.80)[b]	<0.001	64	79	62	81	3.08	0.45
UACR (mg/mg)	0.64 (0.58－0.69)	<0.001	80	48	44	82	1.53	0.42

Abbreviations: AUC, area under curve; CI, confidence interval; Cre, creatinine; NLR, negative likelihood ratio; NPV, negative predictive value; PLR, positive likelihood ratio; PPV, positive predictive value; SEN, sensitivity; SPEC, specificity; UACR, urine albumin-creatinine ratio; uFetA, urinary fetuin-A.

[a] Values were calculated using the Youden's index and the optimal cutoff points (uFetA >110.41 ng/mg Cre and UACR >1.50 mg/mg, respectively), maximizing the sum of sensitivity and specificity.

[b] The AUC of the urinary fetuin-A/creatinine ratio was significantly greater than that of UACR for predicting major adverse kidney events ($P <$ 0.001).

Supplementary Figures

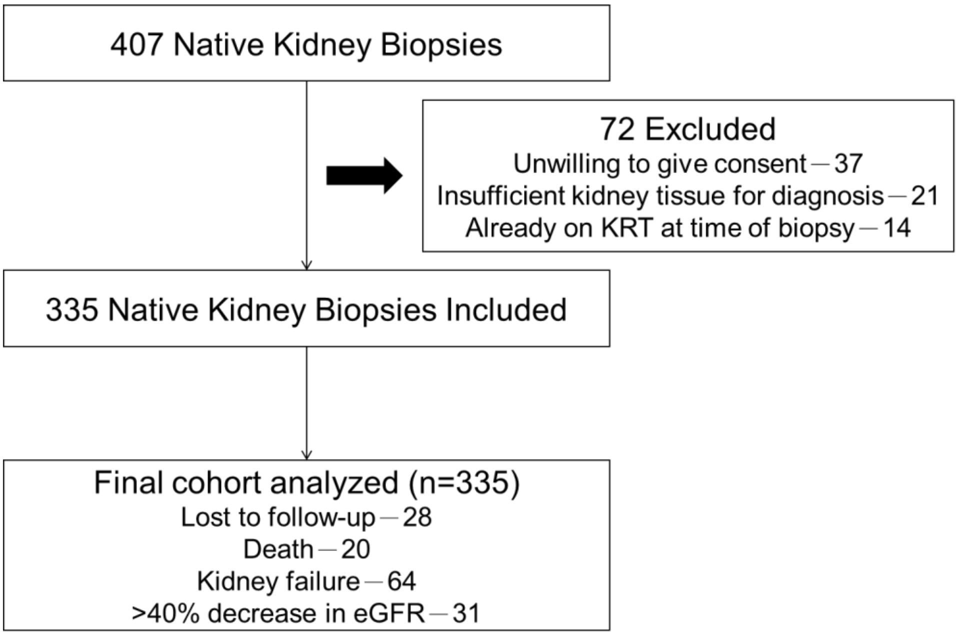

Figure S1. Flow chart of enrollment and exclusion. Abbreviations: eGFR, estimated glomerular filtration rate; KRT, kidney replacement therapy.

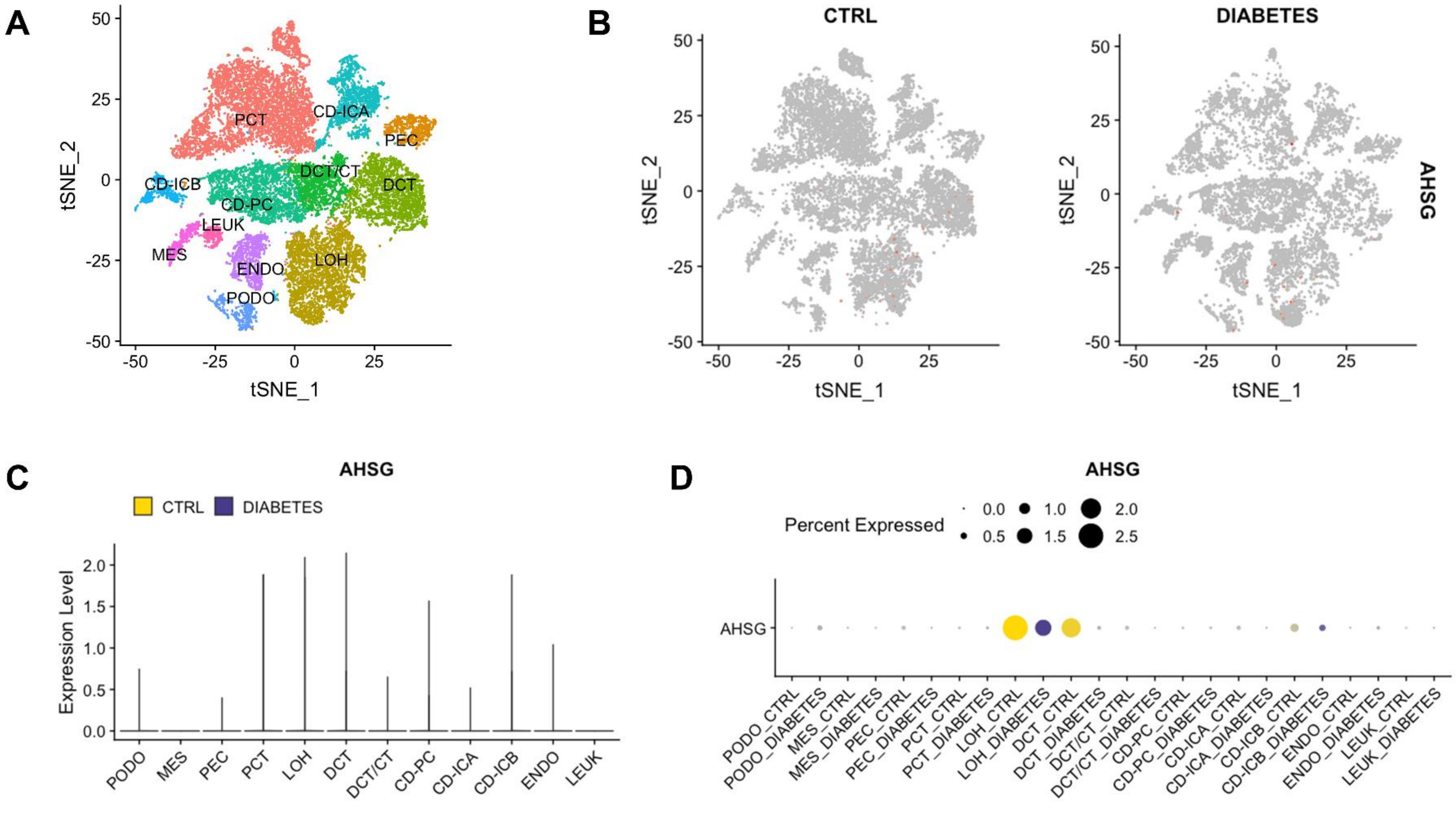

Figure S2. Localization of *AHSG* Expression in the Adult Human Kidney. (A) Based on data from Wilson et al.'s single-nucleus RNA sequencing of human diabetic and control kidney samples, the t-distributed stochastic neighbor embedding (tSNE) projection of 23,980 nuclei from both diabetic and control kidneys reveals 12 distinct cell clusters. (B) t-SNE plots illustrate *AHSG* expression levels in the major cell types found in

control and diabetic kidney disease samples. (C) The violin plot provides insight into the cell-type-specific expression of *AHSG* mRNA. (D) The dot plot showcases the expression of *AHSG* in both control and diabetic kidney samples. (Website: http://humphreyslab.com/SingleCell, accessed on 4 November 2023). Wilson PC, Wu H, Kirita Y, et al. The single-cell transcriptomic landscape of early human diabetic nephropathy. Proc Natl Acad Sci U S A. 2019;116(39):19619-19625.

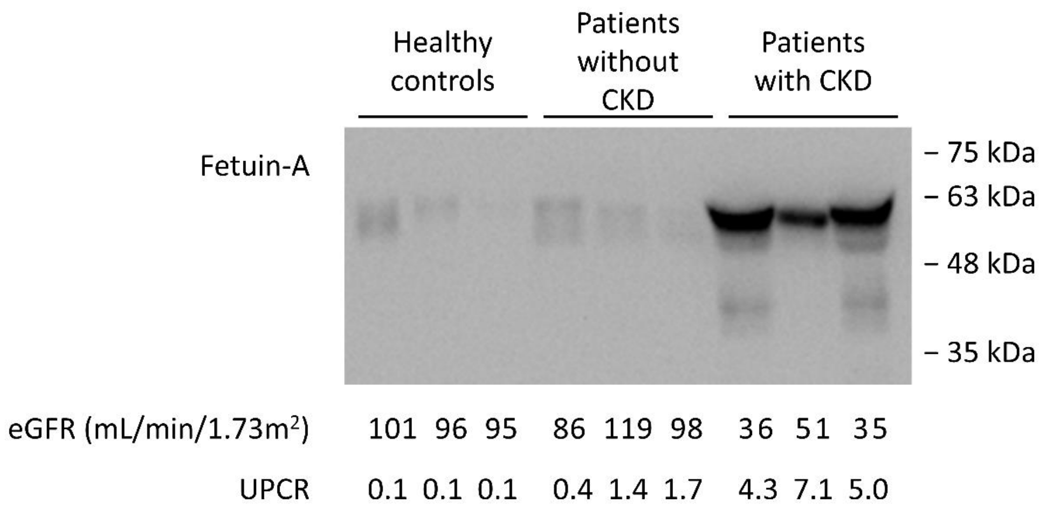

Figure S3. Urinary excretion of fetuin-A in healthy subjects and patients with biopsy-proven kidney disease by Western blotting analysis. Abbreviations: CKD, chronic kidney disease; eGFR, estimated glomerular filtration rate; UPCR, urine protein-to-creatinine ratio.

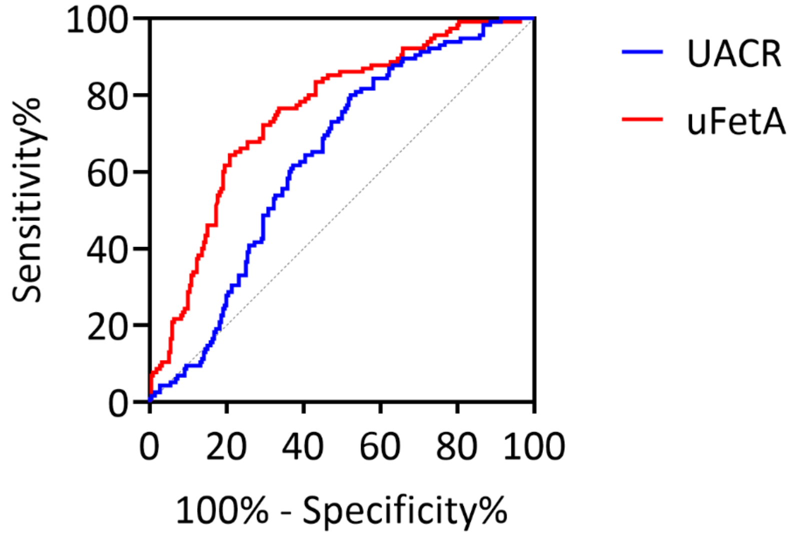

Figure S4. Comparisons of the performance in prognosticating major adverse kidney events between uFetA and UACR were conducted using receiver operating characteristic curve analyses. Abbreviations: UACR, urine albumin-creatinine ratio (mg/mg); uFetA, urinary fetuin-A levels (ng/mg creatinine).
